# Reliability and validity of the Chinese version of the Short Musculoskeletal Function Assessment questionnaire in patients with skeletal muscle injury of the upper or lower extremities

**DOI:** 10.1186/s12891-015-0617-z

**Published:** 2015-07-07

**Authors:** Ying Wang, Zehui He, Lifang Lei, Dingkun Lin, Yajie Li, Gang Wang, Huimin Zhai, Jingli Xu, Guangqing Zhang, Meizhen Lin

**Affiliations:** Department of Orthopedic Trauma, Nan Fang Hospital, Southern Medical University, Guangzhou, 510515 China; Department of Clinical Epidemiology, Second Clinical College, Guangzhou University of Chinese Medicine, Guangzhou, 510120 China; Department of Acupuncture, Second Clinical College, Guangzhou University of Chinese Medicine, Guangzhou, 510120 China; Department of Orthopedic, Second Clinical College, Guangzhou University of Chinese Medicine, Guangzhou, 510120 China; Department of Nursing, Nan Fang Hospital, Southern Medical University, Guangzhou, 510515 China; Department of Humanistic Nursing, College of Nursing, Southern Medical University, Guangzhou, 510515 China; Library, Second Clinical College, Guangzhou University of Chinese Medicine, Guangzhou, 510120 China; Department of Nursing, Second Clinical College, Guangzhou University of Chinese Medicine, Guangzhou, 510120 China

**Keywords:** Musculoskeletal disorders, Chinese, Short Musculoskeletal Function Assessment, Exploratory factor analysis, Quality of life

## Abstract

**Background:**

The Short Musculoskeletal Function Assessment (SMFA) questionnaire is one of the most commonly used scales to evaluate functional status and quality of life (QOL) of patients with a broad range of musculoskeletal disorders. However, a Chinese version of the SMFA questionnaire for the psychometric properties of skeletal muscle injury patients in China is still lacking. The current study translated the SMFA into Chinese and assessed its reliability and validity among Chinese patients with skeletal muscle injury of the upper or lower extremities.

**Methods:**

The original SMFA was translated from English into Chinese and culturally adapted according to cross-cultural adaptation guidelines. A multicenter cross-sectional study was conducted, comprising 339 skeletal muscle injury patients (aged 20–75 years) from 4 hospitals. The SMFA, the health survey short form (SF-36) along with a region-specific questionnaire (including the disabilities of the arm, shoulder, and hand questionnaire (DASH), the hip disability and osteoarthritis outcome score (HOOS), the knee injury and osteoarthritis outcome score (KOOS), and the foot function index (FFI)) were completed according to the region of injury. Reliability was estimated from the internal consistency using Cronbach’s α and validity was assessed via convergent validity, known-groups comparison, and construct validity.

**Results:**

Cronbach’s α coefficient was over 0.75 for two subscales and four categories of the SMFA, suggesting that the internal consistency reliability of the SMFA was satisfactory. Known-groups comparison showed that the dysfunction index and the bother index of the SMFA discriminated well between patients who differed in age, gender, injury location, and operation status rather than in subgroups based on the body mass index (BMI). The convergent validity of the SMFA was good, as moderate to excellent correlations were found between the subscales of the SMFA and the four subscales of SF-36 (physical function, role-physical, bodily pain, and social functioning) and the region-specific questionnaires. The construct validity was proved by the presence of a six-factor structure that accounted for 66.85 % of the variance.

**Conclusion:**

The Chinese version of the SMFA questionnaire is a reliable and valid instrument to measure patient-reported impact of musculoskeletal injuries in the upper or lower extremities.

## Background

Musculoskeletal injuries are a major public health problem because they are one of the main determinants of disability, work absenteeism, and rising health care costs, which can have profound effects on the quality of life (QOL) of the patients [1]. Nevertheless, compared to other countries, function and QOL of patients with musculoskeletal injuries in China has received marginal attention. One of the main reasons for this inattention is the lack of suitable instruments that have been developed or adapted according to established scientific criteria and attributes [2].

The Short Musculoskeletal Function Assessment (SMFA) is one of the patient-reported outcome measures (PROMS) recommended by the American Academy of Orthopedic Surgeons that has been internationally used for more than 14 years for extensive assessment of the functional status of patients with a broad range of musculoskeletal disorders encountered in clinical practice [3–5]. Thus far, the SMFA has been translated, cross-culturally adapted and validated into Swedish [5], German [6], Spanish [4], Brazilian Portuguese [7] and Dutch [8]. However, a Chinese version of the SFMA has not been created as yet.

The aim of this study was to translate the SMFA questionnaire into Chinese, adapt it to cultural specificities, and evaluate the psychometric properties of the Chinese version of the SMFA as expressed by its feasibility, internal consistency, reliability, convergence, and construct validity in patients with skeletal muscle injury of the upper or lower extremities.

## Materials and methods

### Study and design subjects

The study was divided into two stages. In the first stage, the original English version of the SMFA was translated into Chinese. The second stage was a multi-center study, where the reliability and validity of the translated version was evaluated in a cross-sectional study. Patients with skeletal muscle injury of the upper or lower extremities were enrolled from 4 large hospitals between March and September 2014. Inclusion criteria were: upper or lower extremity skeletal muscle injury, age between 20 and 75, fluency in Chinese, and capacity to self-report. Exclusion criteria for this study were: head trauma, spinal injury or fracture with neurological dysfunction, neuromuscular disease, amputation of a limb, cardiovascular disease with an active episode three months prior to the start of this study, cancer, and serious psychiatric or cognitive disorder. Patients with reading or writing disabilities were also excluded.

The study had been filed by Institutional Ethics Committee of Guangdong Provincial Hospital of Traditional Chinese Medicine obtained Ethics review Exemption. Patients provided informed consent prior to their enrollment into the study. The participants were asked to answer the questionnaires according to their own feelings and opinions about limb function, mental states, and daily activities related to their musculoskeletal disorders. After completion, the questionnaires were collected as soon as possible.

### Measurement tools

The measurement tools consisted of a demographic information questionnaire, two QOL ⁄ health status scales (Chinese versions): SMFA, Health Survey Short Form (SF-36), and a region-specific questionnaire (Chinese versions) including the disabilities of the arm, shoulder, and hand questionnaire (DASH), the hip disability and osteoarthritis outcome score (HOOS), the knee injury and osteoarthritis outcome score (KOOS) and the foot function index (FFI), depending on the region of the injury.

The demographic questionnaire gathered socio-demographic information such as age, gender, marital status, height, weight, education level, co-existing chronic diseases, and the clinical type of the musculoskeletal injuries.

The region-specific questionnaires were frequently used for assessment of the local function of the musculoskeletal injuries or disorders, whereas the SF-36 was used to evaluate the convergence validity of the SMFA. The demographic questionnaire was used to evaluate the known-groups comparison of the SMFA.

### SMFA

The SMFA questionnaire was developed by Swiontkowski et al.[3], and concerns the functional and lifestyle disabilities caused by musculoskeletal disorders or injuries. It contains 46 items with 2 subscales, the dysfunction index which has 34 items for the assessment of patient functional performance, and the bother index which has 12 items for the assessment of how much the patients are bothered by their functional problems. The dysfunction index is presented in four categories: the daily activities, emotional status, arm/hand function, and mobility. All items are rated on a 5-point scale with responses of “not at all”, “a little”, “a lot”, “very much” and “impossible” scored as 1, 2, 3, 4, and 5, respectively. The scores for the two parts and the four categories are calculated by summing the responses for the individual items and transforming the scores ranging from zero to 100; higher scores indicate poorer function.

### SF-36

The SF-36 is a generic health status questionnaire that can be used for the evaluation of disease, health status, economic evaluation of population, as well as assessment of the clinical curative effect of the treatment options [9]. The SF-36 Chinese version has been tested for reliability, validity and applicability [10]. It includes 36 items and provides eight scales: physical function (PF), role-physical (RP), bodily pain (BP), general health (GH), vitality (VT), social functioning (SF), role-emotional (RE), and mental health (MH). The scores are calculated according to the scoring algorithm on the SF-36 user manual. The higher the score, the better was the perceived health level.

### Region-specific questionnaires

#### DASH

DASH is a self-administered outcome questionnaire designed to evaluate physical disability and symptoms in people with musculoskeletal disorders of the upper extremity [11, 12]. Its Chinese version has been tested for psychometric properties and is available at http://www.dash.iwh.on.ca. DASH contains 30 items concerning the patient’s health status during the preceding week, including 21 physical function items, 6 symptom items, and 3 social roles/function items. In addition, DASH contains two four-item optional modules: one for the patient’s ability to perform certain motion and/or to play a musical instrument, and the other module for the patient’s ability to work. We only used the 30-item scale in this study.

#### HOOS

HOOS is a simple self-administered questionnaire developed to assess patient opinions regarding hip and related problems, from patients with hip disability with or without osteoarthritis. The Chinese version has been validated for use in China [13]. HOOS consists of 40 items divided into five subscales: pain, other symptoms, functions in activities of daily living (ADL), function in sports and recreation, and hip-related quality of life.

#### KOOS

KOOS is a self-reported joint-specific measure that was developed as an extension of the WOMAC for young and/or active patients with knee osteoarthritis or knee injury [14, 15]. KOOS comprises of 42 items with five subscales: pain, other symptoms, function in ADL, function in sports and recreation, and knee-related quality of life. Its Chinese version has been adapted and tested for the psychometric properties [16].

#### FFI

FFI was developed in 1991 as a patient-reported instrument to measure the impact of foot problems on function in terms of pain and disability [17]. FFI consists of 23 visual analogue scales divided into three subscales: 9 related to pain, 9 related to difficulties, and 5 related to patient limitation. Its Chinese version has been evaluated and has shown good psychometric properties [18].

### Translation process

The process of translation and adaptation into Chinese followed the guidelines recommended by American Academy of Orthopedic Surgeons (AAOS) and the guidelines for cross-cultural adaptation of health-related quality of life measures. Two independent translators with Chinese as their mother tongue (one aware of the concept) translated the American version into Chinese. The two translations were combined into a synthesis and the differences resolved by consensus. Two independent translators with English as their mother tongue then translated this Chinese version of the SMFA back into English. Both translators were blinded to the concepts being investigated and had no medical background. Then, we sent the back translation of SMFA to the original author aiming to test whether it conflicted with the original version. We revised according to the response from the original author, prepared the pre-final version, and tested it on 30 orthopedic outpatients with skeletal muscle injury of the upper or lower extremities before making a few minor adjustments to obtain the final version. The final version of the Chinese SMFA was then used to evaluate its validity and reliability.

### Participants

**Fig. 1:**
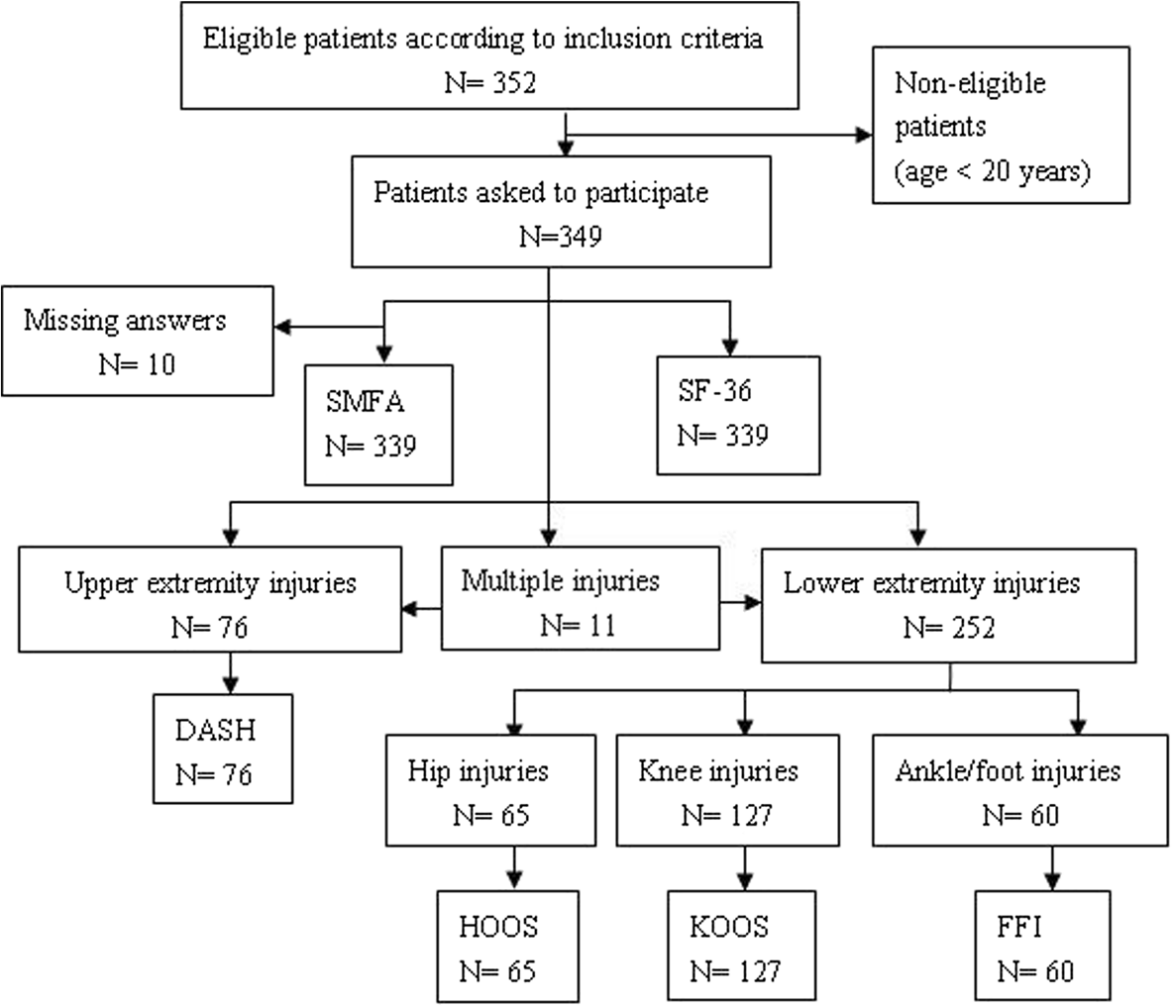
Flow diagram of inclusion of respondentsᅟ

Initially 352 patients were recruited in this study. Out of these, 3 patients were less than 20 years old. Additional 10 patients were excluded because they had missing answers on the SMFA. Finally, the analysis was carried out on a total of 339 patients (96 %). All participants were asked to complete three questionnaires: the SMFA, the SF-36, and a region-specific questionnaire specific to the region of their injury. Of these 339 patients, 76 and 65 patients were asked to complete the DASH and HOOS, respectively, 127 patients filled out the KOOS, and 60 patients filled out the FFI. Other patients with multiple disorders completed the region-specific scales according to their corresponding sites of injury. The flow diagram of the inclusion of respondents is presented in Fig. 1.

### Statistical analysis

For each subscale of the SMFA, the floor and ceiling effects were assessed. These floor and ceiling effects were considered to be present if more than 20 % of respondents achieved the lowest or the highest possible scores [19]. The internal consistency was estimated using Cronbach’s α coefficient. A Cronbach’s α value of more than 0.70 was considered as satisfactory. A known-groups comparison was used to test how well the dysfunction index and bother index of the SMFA discriminated between subgroups of the study sample that differed in their health condition, including age, gender, body mass index (BMI), injury location and operation status. Comparisons were performed by *t*-test or one-way analysis of variance (ANOVA).

The convergence validity of the SMFA was evaluated by assessing the Spearman’s rank correlation coefficients between the SMFA and the SF-36, HOOS, KOOS and FFI, which are the potential measures that assess similar underlying phenomenon as the SMFA. Correlations ranging from 0.25 to 0.50 suggest a fair degree of relationship, those from 0.50 to 0.75 suggest moderate to good relationship, and values greater than 0.75 are considered good to excellent relationships. The construct validity of the SMFA was evaluated by extracting its factor structure using exploratory factor analysis (EFA) with the principal components method. Promax was performed according to the supposed correlations between the factors.

If patients had fewer than 50 % of the answers missing in any category of the dysfunction index, the mean value of that category for the missing item(s) was substituted. When one item of the SMFA from the 35 to 46 items was left unanswered, the questionnaire was excluded from the analysis. Patients who did not complete the demographic questionnaire were excluded.

All statistical analysis was performed using the software SPSS 17.0 for Windows. (SPSS Inc., Chicago, IL, USA).

### Qualitative characteristics

The translation back into English revealed some minor discrepancies that were considered to be related to cultural rather than language differences. The original author approved of the back translation except the question 33 option “You feel emotionally powerless” because the original was “disabled”, which tends to be more about physical function; we revised that item. Furthermore, when testing the revision, many patients reported difficulties understanding the items on the SMFA questionnaire that were specific to American lifestyles. For example, questions that included “Mowing the lawn”, an activity not frequently performed in Chinese families as most families do not have a lawn, needed slight revision. We replaced this activity with the term "moving furniture or heavy items". Additionally, a bathtub is not widely used in China. This limitation was addressed by replacing bathtub with the term “shower” in the Chinese version of the questionnaire.

## Results

A total of 339 patients (mean age 46 years), having various orthopedic injuries and disorders were recruited for this study. More than half of the patients were male (53.1 %), the majority of patients were married or living with a partner (70.2 %), and most had a college or higher education (38.6 %). No participants reported cancer, while 91 (26.8 %) of them reported chronic disease. The most common clinical type of musculoskeletal injury was arthritis (23 %), followed by bruising or trauma (19.2 %), and fracture (8 %). Further demographic and clinical characteristics of the participants are shown in Table 1.

**Table 1:** Demographic and clinical characteristics of the participants

Characteristics	*N* = 339
Age (years) (Mean, SD)	46, 16.0
BMI (Mean, SD)	23.5, 3.6
Gender (%)	
Male	180 (53.1)
Female	159 (46.9)
Marital status (%)	
Single	78 (23.0)
Married or with partner	238 (70.2)
Divorced	12 (3.5)
Widowed	11 (3.2)
Educational level (%)	
Elementary school	114 (33.6)
High school	94 (27.7)
College or higher	131 (38.6)
Other chronic disease (%)	91 (26.8)
Injury location (%)	
Upper limb (Shoulder/Elbow/Arm/wrist/hand)	76 (22.4)
Upper leg/Hip/ Pelvis	65 (19.2)
Lower leg/Knee	127 (37.5)
Ankle/foot	60 (17.7)
Hip and Upper limb	2 (0.6)
Knee and Upper limb	5 (1.5)
Hip and Knee	1 (0.3)
Hip and Knee and Upper limb	1 (0.3)
Knee and Ankle/foot	2 (0.6)
Diagnosis (%)	
Fracture	27 (8.0)
Arthritis	78 (23.0)
Bruise or/and trauma	65 (19.2)
Other	169 (49.9)
Received relevant operation	151 (44.5)

A total of 339 patients were involving in our study. All values, except for age and BMI (mean ± standard deviation), are given as the number of patients, with the percentage in parentheses

There was no ceiling effect for any of the subscales of the SMFA. However, large floor effects were found for the arm and hand function category (36.6 %). The means and standard deviations are presented in Table 2.

**Table 2:** Descriptive statistics of the SMFA scores, internal consistency and floor and ceiling effects

SMFA	No. of items	Mean	SD	% floor	% ceiling	Cronbach’s α	Intraclass correlation
Arm and hand function	8	9.83	14.01	36.6	0	0.870	0.870
Mobility	9	25.50	21.53	14.2	0	0.925	0.925
Daily activities	10	25.81	24.03	6.48	0	0.928	0.928
Emotional status	7	23.38	13.43	4.42	0	0.776	0.776
Dysfunction index	34	21.47	16.17	6.19	0	0.954	0.954
Bother index	12	23.43	18.21	8.85	0	0.935	0.935

*SMFA* Short Musculoskeletal Function Assessment

### Reliability

The Cronbach’s α was 0.954 for the dysfunction index, and 0.935 for the bother index. For the four categories, the Cronbach’s α ranged from 0.776 to 0.928 (Table 2). The internal consistency reliability of the SMFA was found to be satisfactory.

### Validity

#### Known-groups comparison

The known-groups comparison showed that the dysfunction index and the bother index of the SMFA discriminated well between patients who differed in their age, gender, injury location and operation status, but did not discriminate between the subgroups based on the BMI (Table 3).

**Table 3:** The dysfunction index and the bother index score for the patients by age, gender, Body Mass Index, injury location and operation status

	Dysfunction index	*P* value	Bother index	*P* value
	Mean (SD)		Mean (SD)	
Age groups				
18-29 (*n* = 66)	16.24 (15.78)	**<0.001**	18.62 (16.22)	**0.008**
30-44 (*n* = 87)	20.44 (16.27)		22.46 (18.55)	
45-59 (*n* = 96)	20.41 (15.17)		22.81 (18.62)	
≥ 60 (*n* = 79)	27.86 (15.57)		28.74 (18.14)	
Gender				
Male (*n* = 176)	19.18 (16.38)	**0.008**	20.70 (17.62)	**0.005**
Female (*n* = 152)	23.92 (15.48)		26.32 (18.60)	
BMI groups				
Low weight (*n* = 19)	23.80 (20.82)	0.856	28.18 (19.31)	0.294
Normal (*n* = 218)	21.01 (15.85)		23.76 (18.42)	
Overweight (*n* = 57)	21.54 (16.13)		21.24 (16.84)	
Obesity (*n* = 31)	19.85 (14.01)		19.29 (17.99)	
Injury location^a^				
Upper-extremity (*n* = 76)	14.26 (12.47)	**<0.001**	18.89 (16.06)	**0.016**
Lower-extremity^b^ (*n* = 252)	23.52 (16.49)		24.64 (18.71)	
Operation status				
Yes (*n* = 149)	17.36 (15.95)	**<0.001**	19.00 (17.98)	**<0.001**
No (*n* = 179)	24.72 (15.52)		26.89 (17.77)	

Numbers in bold represent *P*-values which are significant at level 0.05

^a^Patients with multiple injuries were excluded (*n* = 11)

^b^Lower-extremity included upper leg/hip/ pelvis, lower leg/knee, and ankle/foot

#### Convergence validity

The correlations between the SMFA categories and the subscales of the SF-36, DASH, HOOS, KOOS, and FFI are presented in Table 4. The results showed moderate to good correlations between the mobility category, dysfunction index and the bother index and the three subscales (PF, RP, and BP) of the SF-36. In 84 patients with upper limb injury, moderate to good correlations were found between the subscales of the SMFA and the DASH. In our study, there were 69 patients with pelvis injury, upper leg or hip disorders, and 136 patients with lower leg or knee disorder. In these patients, the convergent validity was demonstrated through moderate to good correlations between the SMFA category daily activities and two subscales and the ADL subscale of HOOS and KOOS. Only 62 patients were injured in the foot or ankle. The SMFA categories and subscales indices correlated more closely with the limitations of the FFI than foot pain and difficulty with activities, except for emotional status category.

**Table 4:** Spearman’s rank correlation coefficients between the SMFA subscales and SF-36, DASH, HOOS, KOOS, and FFI

	Arm and hand function category	Mobility category	Daily activities category	Emotional status category	Dysfunction index	Bother index
SF-36 (*N* = 339)						
PF	−.344	−.644	−.569	−.527	−.616	−.594
RP	−.364	−.504	−.576	−.530	−.586	−.626
BP	−.353	−.588	−.565	−.613	−.625	−.650
GH	−.267	−.387	−.353	−.540	−.423	−.477
VT	−.290	−.396	−.346	−.544	−.436	−.491
SF	−.393	−.477	−.561	−.536	−.582	−.620
RE	−.310	−.378	−.430	−.492	−.462	−.539
MH	−.183	−.211	−.211	−.446	−.271	−.371
DASH (*N* = 84)						
DASH	.669	n.a.	.730	.560	.750	.695
HOOS (*N* = 69)						
Pain	n.a.	−.652	−.630	−.471	−.628	−.609
Symptoms	n.a.	−.631	−.558	−.416	−.568	−.535
ADL	n.a.	−.728	−.713	−.483	−.738	−.670
Function in sports/recreation	n.a.	−.640	−.627	−.424	−.654	−.540
Quality of life	n.a.	−.680	−.579	−.437	−.620	−.521
KOOS (*N* = 136)						
Pain	n.a.	−.571	−.491	−.472	−.573	−.595
Symptoms	n.a.	−.492	−.380	−.450	−.465	−.499
ADL	n.a.	−.711	−.668	−.444	−.713	−.651
Function in sports/recreation	n.a.	−.626	−.586	−.304	−.608	−.569
Quality of life	n.a.	−.468	−.373	−.346	−.430	−.498
FFI (*N* = 62)						
Foot pain	n.a.	.595	.582	.532	.622	.613
Difficulty with activities	n.a.	.615	.566	.536	.610	.623
Limitations	n.a.	.731	.729	.581	.744	.710

All correlations are significant at the 0.001 level

*n.a.* not applicable, *N* sample size when correlation was calculated, *SMFA* Short Musculoskeletal Function Assessment, *SF-36* Health Survey Short Form, *DASH* Arm, Shoulder, and Hand questionnaire, *HOOS* Hip disability and Osteoarthritis Outcome Score, *KOOS* Knee injury and Osteoarthritis Outcome Score, *FFI* Foot Function Index

The correlation coefficients between SMFA and SF-36, HOOS, and KOOS were negative, because a higher SMFA score indicated greater impairment in QOL, whereas a higher score on SF-36, HOOS, and KOOS indicated better health or performance.

#### Construct validity

The Kaiser–Meyer–Olkin value was 0.953, indicating that the variables were correlated and factor analysis was appropriate for the data set. Using the principal component method, exploratory factor analysis of the SMFA identified a six-factor structure which accounted for 66.852 % of the total SMFA variance.

A six-factor solution with simple structure was also found to be most optimal for the SMFA (Table 5). The first factor was the combination of the daily activities and the mobility categories. The third and fourth factors matched the arm and hand function category and the emotional status categories. The second factor matched the bother index perfectly. The fifth factor was explained by sexual activity and driving, and the sixth factor was explained by difficulties in falling asleep.

**Table 5:** Factor extraction: principal component analysis

	Factor 1	Factor 2	Factor 3	Factor 4	Factor 5	Factor 6
Difficulty to…						
1. Get in or out a low chair	**.784**	.050	−.201	.052	−.119	.261
2. Open bottles	−.153	−.121	**.888**	.129	.008	−.015
3. Shop groceries	**.896**	−.095	−.068	−.071	.229	.062
4. Climb stairs	**1.003**	−.063	−.227	.004	−.016	.040
5. Make a fist	−.259	−.070	**.880**	.092	−.116	.041
6. Use the bathtub or shower	**.697**	−.047	.042	.000	.331	.129
7. Get comfortable to sleep	.242	.031	.122	.104	.094	**.650**
8. Bend or Kneel down	**.860**	−.017	−.194	.064	−.066	.185
9. Use buttons or zippers	−.127	−.005	**.899**	−.015	.041	.088
10. Cut own fingernails	.187	.198	**.524**	−.242	.052	.173
11. Get dressed	.266	.137	**.438**	−.177	.217	.167
12. Walk	**.976**	−.020	−.172	−.048	−.006	.051
13. Move after sitting or lying down	**.751**	.035	−.074	.078	−.189	.246
14. Go out by yourself	**.944**	−.096	−.064	−.023	.165	−.002
15. Drive	.408	−.078	.140	−.010	**.495**	.016
16. Clean yourself after going to the bathroom	**.577**	.008	.215	−.006	.168	.206
17. Turn knobs or levers	.008	−.025	**.796**	.034	−.037	.098
18. Write or type	.008	−.137	**.783**	.191	−.076	−.113
19. Pivot	**.627**	−.037	.070	.100	.133	.291
20. Do your physical recreational activities	**.810**	.048	.004	−.045	.121	−.115
21. Do your leisure activities	**.677**	.048	.178	−.021	.221	−.015
22. Be sexually active	.269	−.023	−.111	.214	**.636**	.095
23. Do light housework	**.580**	.026	.336	−.047	.170	−.117
24. Do heavy housework	**.723**	.069	.207	−.008	.068	−.163
25. Do your usual work	**.643**	.229	.168	−.067	.029	−.197
Frequency…						
26. Walk with a limp	**.653**	.101	−.203	.283	−.054	−.233
27. Avoid using painful limb or back	**.447**	.297	.063	.060	−.148	−.268
28. Leg locked or giving-away	.313	−.120	.085	**.574**	−.184	.178
29. Problems with concentration	−.119	.092	.058	**.722**	.188	.235
30. Doing too much one day affecting what you do the next day	−.046	−.025	.022	**.762**	.253	.087
31. Acting irritated towards those around you	−.221	.122	−.143	**.679**	.410	−.096
32. Being tired	.035	−.044	.139	**.801**	.005	.032
33. Feeling disabled	.177	.039	.202	**.633**	−.027	−.134
34. Feeling angry or frustrated because of injury	.086	.342	.014	**.430**	−.047	−.094
Bothered by…						
35. Problems using arms or legs	.014	**.832**	.139	−.112	−.332	.040
36. Problems using your back	.040	.376	.366	.166	−.270	.042
37. Problems doing chores in and around home	.163	**.672**	.004	.033	.115	−.100
38. Problems with taking care of personal hygiene	.230	**.574**	.079	−.075	.192	−.039
39. Problems with sleep and rest	−.158	**.721**	.005	.091	.048	.393
40. Problems with leisure or recreational activities	.043	**.817**	−.158	.028	.196	.024
41. Problems with important people in your life	−.100	**.797**	−.130	.059	.145	.027
42. Problems with thinking, concentration, or remembering	−.202	**.593**	.022	.272	.132	.144
43. Problems coping with your injury or signs of wear	−.019	**.878**	−.087	−.022	−.241	−.002
44. Problems doing usual work	.129	**.741**	−.159	.059	.210	−.034
45. Problems feeling dependent on others	.007	**.782**	−.069	.018	.170	.015
46. Problems with stiffness and pain	.187	**.749**	.049	−.091	−.313	.054

Substantial (≥0.4) factor loadings are marked bold

## Discussion

The focus of this study was the translation, cross-cultural adaptation and assessment of the reliability and validity of the Chinese version of the SMFA questionnaire among Chinese patients with skeletal muscle injury of the upper or lower extremities. The results confirmed that it is a reliable and valid instrument that can be used in assessing QOL and functional status of Chinese patients with a broad range of musculoskeletal disorders.

To our knowledge, this is the first study in which the floor effect, the ceiling effect and the internal consistency of the four SMFA categories were proved, except for the two subscales. No ceiling effects for any of the SMFA indices and categories were noted. The floor effect, meaning that the patient had the best possible score, was found. A moderate percentage of patients were likely to response “Not at all” in the arm and hand function category, while the floor effects of the two subscales of the SMFA-NL [8], SMFA-BR [7] and the original SMFA [3] were small. However, a floor effect of almost 28 % was reported in the bother Index of the SMFA in patients with femoral neck fractures [20].

The moderate floor effect in the arm and hand function category may be due to our patient population, of which more than 75 % of participants experienced lower limb skeletal muscle injury and were more likely to respond that they had no upper limb function impairment. The internal consistency estimated by Cronbach’s α was excellent in the categories and two indices of the Chinese version of the SMFA, indicating that the instrument has good reliability. Similar results have also been found when verifying other language versions of SMFA [3–7].

Moderate to good correlations between the SMFA and SF-36 and the four region-specific questionnaires added to the available evidence of the convergence validity of the Chinese version of the SMFA. Similar results have been found in the English [3], Swedish [5], Brazilian Portuguese [7], Spanish [4] and Dutch versions [8]. Discrimination in the known-group of the dysfunction index and the bother index of the SMFA were statistically significant based on the age, gender, injury location and operation status, except the BMI group, which were partly consistent with previous studies [3, 21]. In the German version of SMFA, different groups were based on the conservative or operative treatment, pain medication use or ambulatory aid use. So, only discrimination of operation status was consistent with our study [21]. In the original version of SMFA, discrimination was found only in educational level, whereas there were no significant variations in age, gender, and marital status [3]. The difference may be explained by the fact that they performed their analyses on a more heterogeneous population.

Based on the knowledge at the time, the author of the original SMFA hypothesized that the items can be grouped into two subscales and four categories [3]. They utilized three factors, namely, the upper-extremity dysfunction, the lower-extremity dysfunction, and lifestyle dysfunction or bother in the Spanish [4], Brazilian Portuguese version [7], and also in another Dutch version [22] by exploratory factor analysis. There was also a four-factor structure presented in the Dutch version. In our study, however, inconsistent with the existing results, we needed to have six factors. The differences may partly be due to the differences in sample characteristics. In our factor analysis, three items failed to distill into a category or a subscale. These items included sleep, driving, and sexual activity. Sleep did not seem to be associated with the categories such as daily activities, emotional status, arm and hand function, or bother index within our samples. Many problems were encountered regarding the items driving and sexual activity in our study. The patients’ ability to drive a car was found to be conceptually problematic because many people do not drive in China. Sexual activity, on the other hand was a taboo subject for many patients, which may have interfered with their questionnaire responses.

### Strengths and limitations

This is the first study to translate and culturally adapt the SMFA into a Chinese version. The findings, through psychometric evaluation, show that the Chinese version of the SMFA questionnaire is a valid, reliable instrument in assessing the functional status and QOL of patients who have a broad range of musculoskeletal disorders in the upper or lower extremities. Furthermore, this is the first time the floor effect, the ceiling effect and the internal consistency of the four SMFA categories were also proved. Nevertheless, the limitations of our study should be recognized. First, the patients in our study did not include anyone with spine injuries, which may limit the generalizability of using the SMFA in these populations. Secondly, test–retest reliability and responsiveness to change were not evaluated. Finally, the sample size was relatively small, and most of the participants were inpatients and had a lower limb skeletal muscle injury, which might lead to selection bias. Additional studies would include further validation with patients from multi-center clinics, evaluation of test-retest reproducibility and responsiveness of the instruments.

## Conclusion

In summary, the Chinese version of the SMFA is a reliable and valid instrument to measure patient-reported impact of musculoskeletal injuries in the upper or lower extremities.

## Abbreviations

SMFA: Short Musculoskeletal Function Assessment
QOL: Quality of life
SF-36: Health survey short form
DASH: Disabilities of the arm, shoulder, and hand questionnaire
HOOS: Hip disability and osteoarthritis outcome score
KOOS: Knee injury and osteoarthritis outcome score
FFI: Foot function index
BMI: Body mass index
PROMS: Patient-reported outcome measures
PF: Physical function
RP: Role-physical
BP: Bodily pain
GH: General health
VT: Vitality
SF: Social functioning
RE: Role-emotional
MH: Mental health
ADL: Activities of daily living
AAOS: American Association of Orthopedic Surgeons
ANOVA: One-way analysis of variance
EFA: Exploratory factor analysis

## References

[1] Courtney TK, Webster BS (1999). Disabling occupational morbidity in the United States. An alternative way of seeing the Bureau of Labor Statistics’ data. J Occup Environ Med.

[2] Aaronson N, Alonso J, Burnam A, Lohr KN, Patrick DL, Perrin E, Stein RE (2002). Assessing health status and quality-of-life instruments: attributes and review criteria. Qual Life Res.

[3] Swiontkowski MF, Engelberg R, Martin DP, Agel J (1999). Short Musculoskeletal Function Assessment questionnaire: validity, reliability, and responsiveness. J Bone Joint Surg Am.

[4] Guevara CJ, Cook C, Pietrobon R, Rodriguez G, Nunley J, Higgins LD, Olson SA, Vail TP (2006). Validation of a Spanish version of the Short Musculoskeletal Function Assessment Questionnaire (SMFA). J Orthop Trauma.

[5] Ponzer S, Skoog A, Bergstrom G (2003). The Short Musculoskeletal Function Assessment Questionnaire (SMFA): cross-cultural adaptation, validity, reliability and responsiveness of the Swedish SMFA (SMFA-Swe). Acta Orthop Scand.

[6] Bohm TD, Kirschner S, Kohler M, Wollmerstedt N, Walther M, Matzer M, Faller H, Konig A (2005). The German Short Musculoskeletal Function Assessment questionnaire: reliability, validity, responsiveness, and comparison with the Short Form 36 and Constant score--a prospective evaluation of patients undergoing repair for rotator cuff tear. Rheumatol Int.

[7] Taylor MK, Pietrobon R, Menezes A, Olson SA, Pan D, Bathia N, DeVellis RF, Kume P, Higgins LD (2005). Cross-cultural adaptation and validation of the Brazilian Portuguese version of the short musculoskeletal function assessment questionnaire: the SMFA-BR. J Bone Joint Surg Am.

[8] Reininga IH, el Moumni M, Bulstra SK, Olthof MG, Wendt KW, Stevens M (2012). Cross-cultural adaptation of the Dutch Short Musculoskeletal Function Assessment questionnaire (SMFA-NL): internal consistency, validity, repeatability and responsiveness. Injury.

[9] Ware JESK, Kosinski MK, Gandek B (1993). SF-36 Health Survey: Manual and Interpretation Guide.

[10] Li LWH, Shen Y (2003). Chinese SF-36 Health Survey: translation, cultural adaptation, validation, and normalisation. J Epidemiol Community Health.

[11] Hudak PL, Amadio PC, Bombardier C (1996). Development of an upper extremity outcome measure: the DASH (disabilities of the arm, shoulder and hand) [corrected]. The Upper Extremity Collaborative Group (UECG). Am J Ind Med.

[12] Alotaibi NM (2008). The cross-cultural adaptation of the disability of arm, shoulder and hand (DASH): a systematic review. Occupational therapy international.

[13] Wei X, Wang Z, Yang C, Wu B, Liu X, Yi H, Chen Z, Wang F, Bai Y, Li J (2012). Development of a simplified Chinese version of the hip disability and osteoarthritis outcome score (HOOS): cross-cultural adaptation and psychometric evaluation. Osteoarthritis Cartilage.

[14] Roos EM, Roos HP, Ekdahl C, Lohmander LS (1998). Knee injury and Osteoarthritis Outcome Score (KOOS)--validation of a Swedish version. Scand J Med Sci Sports.

[15] Roos EM, Roos HP, Lohmander LS, Ekdahl C, Beynnon BD (1998). Knee Injury and Osteoarthritis Outcome Score (KOOS)--development of a self-administered outcome measure. J Orthop Sports Phys Ther.

[16] Xie F, Li SC, Roos EM, Fong KY, Lo NN, Yeo SJ, Yang KY, Yeo W, Chong HC, Thumboo J (2006). Cross-cultural adaptation and validation of Singapore English and Chinese versions of the Knee injury and Osteoarthritis Outcome Score (KOOS) in Asians with knee osteoarthritis in Singapore. Osteoarthritis Cartilage.

[17] Budiman-Mak ECK, Roach KE (1991). The Foot Function Index: a measure of foot pain and disability. J Clin Epidemiol.

[18] Wu S-H, Liang H-W, Hou W-H (2008). Reliability and validity of the Taiwan Chinese version of the foot function index. J Formos Med Ass.

[19] Anderson RT, Rajagopalan R (1997). Development and validation of a quality of life instrument for cutaneous diseases. J Am Acad Dermatol.

[20] Hedbeck CJTJ, Ponzer S, Blomfeldt R, Bergström G (2011). Responsiveness of the Short Musculoskeletal Function Assessment (SMFA) in patients with femoral neck fractures. Qual Life Res.

[21] Wollmerstedt N, Kirschner S, Faller H, Konig A (2006). Reliability, validity and responsiveness of the German Short Musculoskeletal Function Assessment Questionnaire in patients undergoing surgical or conservative inpatient treatment. Quality of life research : an international journal of quality of life aspects of treatment, care and rehabilitation.

[22] Van Son MA, Den Oudsten BL, Roukema JA, Gosens T, Verhofstad MH, De Vries J (2014). Psychometric properties of the Dutch Short Musculoskeletal Function Assessment (SMFA) questionnaire in patients with a fracture of the upper or lower extremity. Qual Life Res.

